# A comparative pharmacokinetic study of PARP inhibitors demonstrates favorable properties for niraparib efficacy in preclinical tumor models

**DOI:** 10.18632/oncotarget.26354

**Published:** 2018-12-14

**Authors:** Kaiming Sun, Keith Mikule, Zebin Wang, Grace Poon, Aparajitha Vaidyanathan, Gillian Smith, Zhi-Yi Zhang, Jeffrey Hanke, Sridhar Ramaswamy, Jing Wang

**Affiliations:** ^1^ TESARO Inc, Waltham, MA, USA; ^2^ Division of Cellular Medicine, School of Medicine, University of Dundee, Jacqui Wood Cancer Centre, Ninewells Hospital & Medical School, Dundee, UK

**Keywords:** niraparib, tumor exposure, brain exposure, *BRCA*wt tumor, intracranial tumor

## Abstract

Niraparib is an orally bioavailable and selective poly (ADP-ribose) polymerase (PARP)-1/-2 inhibitor approved for maintenance treatment of both *BRCA* mutant (mut) and *BRCA* wildtype (wt) adult patients with recurrent epithelial ovarian, fallopian tube, or primary peritoneal cancers who have demonstrated a complete or partial response to platinum-based chemotherapy. In patients without germline *BRCA* mutations (non-g*BRCA*mut), niraparib improved progression-free survival (PFS) by 5.4 months, whereas another PARP inhibitor (PARPi) olaparib supplied only 1.9 months of improvement in a similar patient population. Previous studies revealed higher cell membrane permeability and volume of distribution (V_D_) as unique features of niraparib in comparison to other PARPi including olaparib. Here, we explore the potential correlation of these pharmacokinetic properties to preclinical antitumor effects in *BRCA*wt tumors. Our results show that at steady state, tumor exposure to niraparib is 3.3 times greater than plasma exposure in tumor xenograft mouse models. In comparison, the tumor exposure to olaparib is less than observed in plasma. In addition, niraparib crosses the blood-brain barrier and shows good sustainability in the brain, whereas sustained brain exposure to olaparib is not observed in the same models. Consistent with its favorable tumor and brain distribution, niraparib achieves more potent tumor growth inhibition than olaparib in *BRCA*wt models and an intracranial tumor model at maximum tolerated doses (MTD). These findings demonstrate favorable pharmacokinetic profiles and potent antitumor effects of niraparib in *BRCA*wt tumors, consistent with its broader clinical effect in patients with both *BRCA*mut and *BRCA*wt tumors.

## INTRODUCTION

PARP1 and 2 are key enzymes acting as DNA damage sensors and signal transducers in response to DNA damage. PARP binds to DNA sites with single-strand breaks (SSBs), catalyzes the poly(ADP-ribosyl)ation (PARylation) of target proteins and recruits DNA repair proteins to these sites [1–4]. Consistent with the importance of PARP in DNA repair, inhibition of PARP activity in homologous recombination (HR)-deficient cells (such as those with *BRCA* and *PALB2* mutations) induces cell death due to a mechanism known as ‘synthetic lethality’, as it is difficult for cancer cells to tolerate simultaneous loss of both PARP-dependent SSB repair and HR-mediated double-strand break (DSB) repair machinery. This important observation led to the pharmaceutical development of PARP inhibitors (PARPi) [5–7]. Currently, at least 6 PARPi are being evaluated actively in clinical settings in a variety of cancer types.

Clinical development of PARPi is most advanced in ovarian cancers (OC), the most common cause of gynecologic cancer death in the United States with 22,000 newly diagnosed cases each year [8]. The majority of High Grade Serous Ovarian Cancer (HGSOC), the most aggressive subtype of OC, are characterized by DNA repair deficiency. Approximately 40-50% of HGSOC have some level of genetic or epigenetic alterations in HR repair pathways including mutations in *BRCA1/2*, *Fanconi anemia* genes, core HR genes, DNA damage response genes and epigenetic silencing of *BRCA1* via promoter hypermethylation [9–16]. In addition, about one-third of HGSOC cases are possibly HR deficient with alterations in non-*bona fide* HR genes, such as *PTEN* loss, *EMSY* amplification, *CDK12* mutations, and overexpression of specific miRNAs [9, 17–21]. Therefore, it has been estimated that only approximately 15% of HGSOC, such as cancers with *cyclin E* amplification, are HR proficient [16]. Even though the majority of HGSOC are considered HR deficient, individual sensitivities to PARPi are different, perhaps due to differing degrees of HR deficiency influenced by genetic and epigenetic alterations. For example, Murai and colleagues showed that *BRCA1/2* or *XRCC2/3* mutant cells are more sensitive to PARPi treatment *in vitro* than *RAD54, FANCC,* or *POLH/Z* mutant cells by 1 to 2 orders of magnitude [22], indicating that for a PARPi to be efficacious, higher concentration of drug is required in *BRCA*wt than *BRCA*mut tumors.

Olaparib is the first FDA approved PARPi for *BRCA*mut OC, whereas niraparib is the first PARPi demonstrating clinical effect in both *BRCA*wt and *BRCA*mut OC, and approved for maintenance treatment based on data from the large Phase 3, double-blinded NOVA trial enrolled patients with recurrent OC responsive to platinum therapies. In the NOVA trial, not only the median duration of progression-free survival (mPFS) improved by 15.5 months (from 5.5 to 21.0 months) in patients with germline *BRCA* (g*BRCA*) mutation, but the mPFS of patient without g*BRCA* mutation also improved by 5.4 months (from 3.9 to 9.3 months) [23]. In a similar population of *BRCA*wt patients, olaparib provided a 1.9-month improvement in mPFS (from 5.5 to 7.4 months) in a randomized Phase 2 trial, Study 19 [24]. The mechanism of action of these two PARPi, which could potentially explain the different clinical effects in *BRCA*wt OC, has yet to be explored.

The anti-tumor effect of PARPi has been attributed to their ability to inhibit DNA repair and to trap PARP on DNA and ultimately induce DSB formation, with the former mechanism thought to be more HR deficiency-specific [22, 25–28]. The PARP inhibition potency and PARP trapping ability of niraparib and olaparib are similar in cell-free systems established using recombinant proteins [22, 26, 29]. In cultured cancer cells, niraparib is approximately twice as potent as olaparib in trapping PARP onto DNA [22, 30]. Similarly, their abilities to induce S-phase-specific DNA damage response and cancer cell death are also different with niraparib being a few fold more potent than olaparib in both *BRCA* mutant and wildtype cells [22, 30–32]. One potential explanation is different cell membrane permeabilities of the two drugs, as the apparent permeability coefficient (Papp) of niraparib (12 to 18 × 10^6^ cm/s) is higher than that of olaparib (3 to 9 × 10^6^ cm/s) [33, 34]. In addition to low permeability, olaparib has a very low solubility in aqueous solutions and therefore is classified as Class IV drug according to the Biopharmaceutical Classification System (BCS)[35, 36], whereas niraparib is a BCS Class I (high permeability and high solubility) or Class II (high permeability and low solubility) drug when administered at 200 or 300 mg in humans, respectively [34]. The volume of distribution (V_D_) for niraparib (≈1220 L) is also higher than that of olaparib (≈158 L) in humans at steady state [37, 38], indicating a potential higher tendency of niraparib to concentrate in the peripheral body compartment including solid tumors rather than in plasma. Indeed, in a Phase 1 study of 60 patients with invasive breast cancer, the average tumor concentration of olaparib was 41% of the plasma concentration [39], potentially attributed to its low V_D_. The tumor exposure to niraparib has not yet been reported in clinical settings.

To explore whether the biophysical properties intrinsic to niraparib, such as high permeability and V_D_, may contribute to its broader clinical activity in patients with or without *BRCA* mutations, the pharmacokinetic profiles and efficacies of niraparib and olaparib were compared in preclinical tumor models. Our results show that niraparib tumor exposure is significantly higher than plasma exposure, which is consistent with its high V_D_. In comparison, olaparib tumor exposure is lower than plasma exposure. In addition, niraparib permeates the brain, whereas olaparib shows very limited brain exposure at maximum tolerated dose (MTD). Importantly, in *BRCA*wt tumor xenograft models and an intracranial tumor model, niraparib achieved more potent tumor growth inhibition than olaparib.

## RESULTS

### Niraparib preferentially concentrates in tumor compared to plasma and penetrates the blood-brain barrier

To understand the difference in pharmacokinetic profiles, exposure to niraparib and olaparib was evaluated in tumor, plasma and other body tissues collected from both ovarian and breast cancer xenograft models. The treatment doses and schedules were defined based on the MTD in each model, as previously reported [40] or determined in the models used in this study.

**Figure 1:**
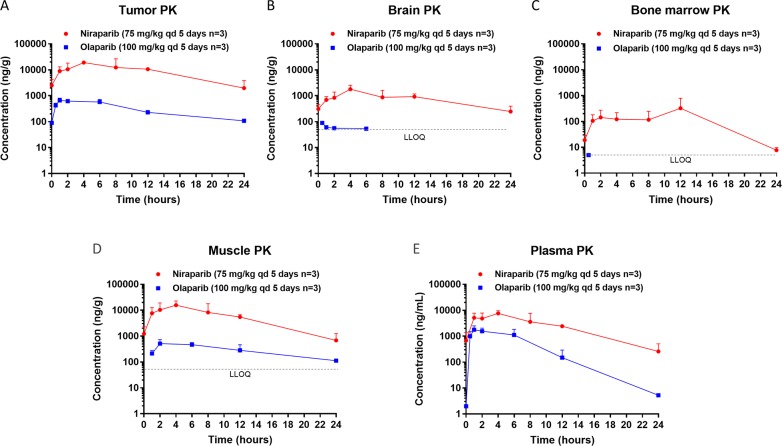
Steady state pharmacokinetics (PK) of niraparib and olaparib in tumor, brain, bone marrow, muscle, and plasma **(A)** Tumor, **(B)** Brain, **(C)** Bone marrow, **(D)** Muscle, and **(E)** Plasma PK in the *BRCA1*mut MDA-MB-436 TNBC xenograft model treated with niraparib or olaparib at the maximum tolerated dose. Niraparib and olaparib were administered orally at 75 and 100 mg/kg qd, respectively. All mice were treated for 5 days, and samples were collected on the last day of treatment at pre-dose and 1, 2, 4, 8, 12, and 24 hours post dose for niraparib and at pre-dose and 0.5, 1, 2, 6, 12, and 24 hours post dose for olaparib. LLOQ=lower limit of quantification.

Tumor, brain, bone marrow, muscle, and plasma exposure to niraparib and olaparib were measured in samples collected from NOD/SCID mice orthotopically inoculated with *BRCA1*mut MDA-MB-436 triple-negative breast cancer (TNBC) cells and treated with 75 mg/kg of niraparib once daily (qd) or olaparib (100 mg/kg qd) for 5 days. The time of maximum concentration observed (t_max_), observed mean of maximum concentration (C_max_) and the WinNonlin calculated area under the curve (AUC_0-24h_) of niraparib at steady state were all significantly higher than those of olaparib in all tissue types tested, despite the fact that the daily dose of niraparib was lower in this *BRCA1*mut model (Figure 1A–1E; Table 1). The dose-normalized exposure to niraparib was 10-, 51-, and 100-fold higher than that to olaparib in plasma, tumor, and brain, respectively (Table 1).

**Table 1:** Steady State Tissue and Plasma PK of Niraparib and Olaparib in MDA-MB-436 TNBC Model

PARPi	Tumor PK				Brain PK				Bone Marrow PK				Muscle PK				Plasma PK			
	C_max_(μg/g)	t_max_(h)	AUC_0-24h_(μg/g·h)	Dose normalized AUC_0-24h_(μM·h/mg)	C_max_(μg/g)	t_max_(h)	AUC_0-24h_(μg/g·h)	Dose normalized AUC_0-24h_(μM·h/mg)	C_max_(μg/g)	t_max_(h)	AUC_0-24h_(μg/g·h)	Dose normalized AUC_0-24h_(μM·h/mg)	C_max_(μg/g)	t_max_(h)	AUC_0-24h_(μg/g·h)	Dose normalized AUC_0-24h_(μM·h/mg)	C_max_(μg/mL)	t_max_(h)	AUC_0-24h_(μg/mL·h)	Dose normalized AUC_0-24h_(μM·h/mg)
Niraparib(75 mg/kg qd 5 days)	19.10	4	213.96	8.90	1.78	4	18.72	0.78	0.32	12	2.82	0.12	15.63	4	139.80	5.82	7.62	4	65.08	2.71
Olaparib(100 mg/kg qd 5 days)	0.66	1	7.59	0.17	0.09	0.5	0.34	0.01	LLOQ	ND	LLOQ	ND	0.51	1	3.29	0.08	1.77	1	11.28	0.26
Dose normalizedAUC_nir/ola_ Ratio	-	-	-	51	-	-	-	100	-	-	-	-	-	-	-	77	-	-	-	10

AUC_0–24h_=area under the plasma concentration–time curve from 0 to 24 hours; LLOQ=lower limit of quantification; ND= not determined; nir=niraparib; ola=olaparib.

Although tissue distribution of a drug often largely depends on its physicochemical characteristics, the abnormal vasculature, reactive stroma, and inflammation that characterize the tumor microenvironment may affect drug penetration [41]. Therefore, tissue and plasma exposure to niraparib and olaparib were also measured in samples collected from BALB/c nude mice subcutaneously implanted with the *BRCA*wt OVC134 ovarian tumor fragment and treated with niraparib (50 mg/kg qd) or 67 mg/kg of olaparib twice a day (bid) for 2 days at MTDs previously determined in this model. Similar to results obtained from the *BRCA*mut TNBC xenograft model, niraparib demonstrated superior exposure over olaparib in all tissue types tested in this *BRCA*wt ovarian cancer xenograft model, although again niraparib was administered with a lower daily dose than olaparib (Supplementary Figure 1A–1C; Table 2). The dose-normalized exposure to niraparib was 3-, 16-, and 34-fold higher than that to olaparib in plasma, tumor, and brain, respectively (Table 2). The results from both xenograft models consistently demonstrate significantly higher exposure to niraparib in all tissue types tested including plasma, tumor, and brain regardless of the differences in *BRCA* status, tumor type or host strain of mice.

**Table 2:** Tissue and Plasma PK of Niraparib and Olaparib in OVC134 Ovarian PDX Model

PARPi	Tumor PK				Brain PK				Plasma PK			
	C_max_(μg/g)	t_max_(h)	AUC_0-last_(μg/g·h)	Dose normalizedAUC_0-24h_ (μM·h/mg)	C_max_(μg/g)	t_max_(h)	AUC_0-last_(μg/g·h)	Dose normalizeAUC_0-24h_ (μM·h/mg)	C_max_(μg/mL)	t_max_(h)	AUC_0-last_(μg/mL·h)	Dose normalizedAUC_0-24h_ (μM·h/mg)
Niraparib(50 mg/kg qd 2 days)	10.80	4	83.14	5.19	0.65	4	8.31	0.52	3.24	4	25.50	1.59
Olaparib(67mg/kg bid 2 days)	6.17	0.5	9.73	0.33	0.52	0.5	0.45	0.02	15.04	0.5	15.59	0.53
Dose normalizedAUC_nir/ola_ ratio	-	-	-	16	-	-	-	34	-	-	-	3

AUC_0–last_=area under the plasma concentration–time curve from 0 to the time of the last PK sampling post-dose, for niraparib AUC_0–last_ is AUC_0–24h_, for olaparib AUC_0–last_ is AUC_0–12h_; nir=niraparib; ola=olaparib.

**Figure 2:**
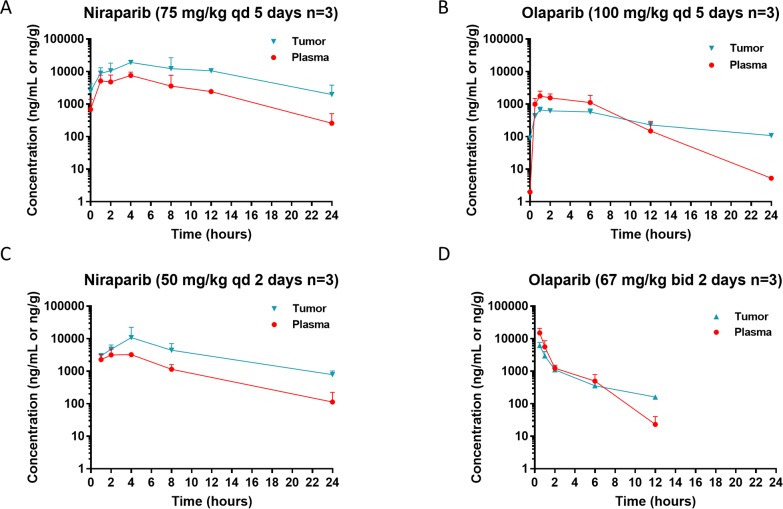
Steady state pharmacokinetics (PK) of niraparib and olaparib in tumor vs plasma Tumor and plasma PK in the *BRCA1*mut MDA-MB-436 TNBC cell line-derived xenograft model **(A & B)** and *BRCA*wt OVC134 ovarian cancer PDX model **(C & D)** treated with niraparib (A & C) or olaparib (B & D) at the maximum tolerated dose. Niraparib and olaparib were administered at 75 or 100 mg/kg once daily, respectively, in MDA-MB-436 model and 50 mg/kg once daily or 100 mg/kg twice daily, respectively in OVC134 model. All mice from MDA-MB-436 model were treated for 5 days, and samples were collected on the last day of treatment at pre-dose and 1, 2, 4, 8, 12, and 24 hours post dose for niraparib and at pre-dose and 0.5, 1, 2, 6, 12, and 24 hours post dose for olaparib. All mice from OVC134 model were treated for 2 days, and samples were collected on the last day of treatment at 1, 2, 4, 8, and 24 hours for niraparib and at 0.5, 1, 2, 6, and 12 hours for olaparib.

The tissue distribution of niraparib and olaparib were also compared in both the MDA-MB-436 and OVC134 models. After 5-day treatment in the MDA-MB-436 model or 2-day treatment in the OVC134 model, niraparib tumor exposure was 3.3-fold of plasma exposure (Figure 2A & 2C and Table 3), suggesting that niraparib tends to concentrate in tumor relative to plasma. In comparison, the tumor exposure to olaparib was only 0.6- to 0.7-fold of plasma exposure in the same studies regardless of the once daily or twice daily dosing schedule (Figure 2B & 2D and Table 3).

**Table 3:** Tissue Distribution of Niraparib and Olaparib

Model	PARPi	Tumor AUC_0-last_(μg/g·h)	Brain AUC_0-last_(μg/g·h)	Bone MarrowAUC_0-last_ (μg/g·h)	Plasma AUC_0-last_(μg/mL·h)	AUC_0-last_Tumor:Plasma	AUC_0-last_Brain:Plasma	AUC_0-last_Tumor:Bone Marrow
MDA-MB-436	Niraparib(75 mg/kg qd 5 days)	213.96	18.72	2.82	65.08	3.3	0.29	75.9
	Olaparib(100 mg/kg qd 5 days)	7.59	0.34	LLOQ	11.28	0.7	0.03	ND
OVC134	Niraparib(50 mg/kg qd 2 days)	83.14	8.31	-	25.50	3.3	0.33	-
	Olaparib(67 mg/kg bid 2 days)	9.73	0.45	-	15.59	0.6	0.03	-

AUC_0–last_=area under the plasma concentration–time curve from 0 to the time of the last PK sampling post-dose, for niraparib AUC_0–last_ is AUC_0–24h_, for olaparib AUC_0–last_ is AUC_0–24h_ in MDA-MB-436 model and AUC_0–12h_ in OVC134 model; LLOQ=lower limit of quantification; ND= not determined.

The brain exposure to these 2 drugs was also significantly different. In both tumor models, the brain to plasma exposure (AUC) ratio of niraparib was ≈0.3, whereas that of olaparib was ≈0.03 (Table 3). While the exposure to niraparib in brain was highly sustainable, olaparib was detectable in brain only for the first 6 hours following drug administration in both MDA-MB-436 (Figure 1B) and OVC134 (Supplementary Figure 1B) models. Because the brain exposure to a drug is regulated by ABC (efflux) transporters, such as BCRP or P-glycoprotein (P-gp, MDR1) which are highly expressed at the blood-brain barrier (BBB), the efflux rates of both PARPi were compared in cells overexpressing these transporters, to understand the potential cause of different brain exposures. Although both niraparib and olaparib are substrates of P-gp [38, 42, 43], the net efflux ratios of niraparib were approximately 2 to 5 times lower than those of olaparib at 3 different concentrations tested in the MDCKII cells overexpressing BCRP or P-gp (Supplementary Table 1). The low efflux rate of niraparib in this *in vitro* assay, which could potentially be attributed to its high bio-membrane permeability, may explain why niraparib could overcome ABC transporter-mediated efflux at the BBB and achieve significantly higher brain exposure than olaparib.

Together, these results demonstrate unique properties of niraparib including its ability to concentrate in tumor relative to plasma and to permeate the BBB, consistent with its high V_D_. Moreover, studying tumor exposure in addition to plasma exposure revealed profoundly distinct pharmacokinetic profiles *in vivo* for drugs of the same class.

### Niraparib induces more potent tumor growth inhibition than olaparib in some *BRCA*wt tumor models

Because MTD administration of niraparib achieved significantly higher exposure in tumor than olaparib, we next evaluated whether there was any association between tumor exposure to PARPi and its growth inhibition. To compare the antitumor activities of niraparib and olaparib, these PARPi were evaluated in 2 commonly used ovarian cancer or breast cancer cell line-derived xenograft (CDX) models and 4 patient-derived xenograft (PDX) models.

**Figure 3:**
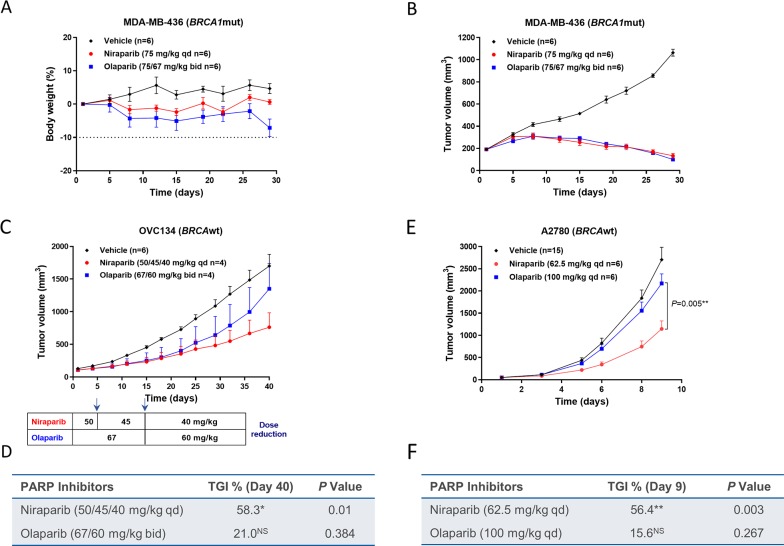
Effect of niraparib and olaparib on tumor volume and body weight in a *BRCA1*mut MDA-MB-436 TNBC cell line-derived xenograft model **(A & B)**, *BRCA*wt OVC134 ovarian cancer PDX model **(C & D)**, and *BRCA*wt A2780 ovarian cancer cell line-derived xenograft model **(E & F)**. (A) Percentage change in body weight and (B) tumor volume of the MDA-MB-436 model treated with niraparib or olaparib at the maximum tolerated dose. Niraparib was administered at 75 mg/kg daily for 28 days. Olaparib was administered at 75 mg/kg twice daily for 7 days and then at 67 mg/kg twice daily for 21 days due to significant body weight loss observed in the olaparib-treated group. (C) Tumor growth of the OVC134 model treated with niraparib or olaparib at the maximum tolerated dose. Dose reductions due to body weight loss are indicated in the chart below the growth curve for both the niraparib- and olaparib-treated groups. Two mice from each treated group were given extensive dose holidays and therefore were excluded from final analysis (D) Table summarizing TGI and *P* value calculated by Student’s *t* test for niraparib or olaparib compared to vehicle on day 40, ^*^P<0.05, NS=not significant. (E) Tumor growth of A2780 xenograft model treated with niraparib or olaparib at the maximum tolerated dose. Niraparib and olaparib were administered at 62.5 and 100 mg/kg daily, respectively. *P* value calculated by Student’s *t* test to compare niraparib and olaparib on day 9. (F) Table summarizing TGI and *P* value calculated by Student’s *t* test for niraparib or olaparib compared to vehicle on day 9, ^**^P< 0.01, NS=not significant.

The *BRCA1*mut MDA-MB-436 TNBC CDX model was treated with MTDs of niraparib or olaparib, with niraparib administered orally at 75 mg/kg qd for 28 days and olaparib administered orally at 75 mg/kg bid for 7 days and then reduced to 67 mg/kg bid for the last 21 days due to body weight loss (Figure 3A). Both niraparib and olaparib achieved a similar degree of tumor regression in this model (Figure 3B), suggesting that the tumor exposure to both PARPi are above the exposure required to achieve their full anti-tumor response in this *BRCA*mut tumor model. Because *BRCA*mut tumors are highly sensitive to PARPi as previously reported in cultured cells [22], the required exposure to PARPi in the *BRCA*mut tumors may be lower than that in the less sensitive tumors. For this reason, we hypothesized that differences in efficacy might be revealed in *BRCA*wt models, where higher drug concentrations are required to induce tumor cell death.

To test this hypothesis, the antitumor effects of niraparib and olaparib were evaluated in 5 *BRCA*wt tumor models. The *BRCA*wt OVC134 ovarian cancer PDX model was treated with MTDs of niraparib or olaparib, with niraparib initially administered at 50 mg/kg and reduced to 40 mg/kg qd, and olaparib first administered at 67 mg/kg and reduced to 60 mg/kg bid, both due to body weight loss. Niraparib markedly reduced tumor growth with 58% tumor growth inhibition (TGI), whereas olaparib treatment had little effect (Figure 3C, 3D, & Supplementary Figure 2A).

The antitumor activities of niraparib and olaparib were then examined in the *BRCA*wt A2780 ovarian cancer CDX model, with niraparib and olaparib administered at 62.5 and 100 mg/kg once daily, respectively. Neither drug caused severe body weight loss or noticeable toxicity in this model (data not shown). Even though in cultured A2780 cells, the cytotoxicities induced by niraparib and olaparib were not statistically different (Supplementary Figure 3), the *in vivo* efficacy of these two PARPi were significantly different (*P* = 0.005) in this ovarian cancer CDX model established with the same cell line (Figure 3E). Niraparib treatment induced significant tumor growth inhibition (TGI = 56.4%), whereas the effect of olaparib was minimal (TGI = 15.6%) and nonsignificant (Figure 3F, & Supplementary Figure 2B). One potential explanation of these results is that the tumor concentration of a drug is relatively more important when the sensitivity to the drug is moderate, such as in the *BRCA*wt OVC-134 and A2780 tumors.

The antitumor activities of niraparib and olaparib were also examined in 3 additional *BRCA*wt ovarian cancer models, OVC527, OV5308 and OVCAR3, where both drugs showed similar degree of efficacy (Supplementary Figure 4). In OVC527 and OV5308 models, both niraparib and olaparib induced similar degrees of tumor regression, suggesting that, similar to *BRCA*mut tumors, these *BRCA*wt but HR deficient tumors are also highly sensitive to PARPi. In contrast, both drugs had minimum antitumor effects in the OVCAR3 CDX model, perhaps due to lack of HR deficiency or inherent resistance to PARPi.

To better understand the pharmacodynamic effects of these PARPi in tumor, we attempted to correlate PARylation inhibition with antitumor effects in preclinical models. However, potent total PARylation inhibition (>85-90% inhibition compared to vehicle control) was always observed over time regardless of efficacy for both PARPi in *BRCA*mut MDA-MB-436 and *BRCA*wt OVC134 models (data not shown), suggesting that inhibition of total PARylation may not be a good biomarker for PARPi efficacy.

Taken together, these results demonstrate the antitumor effect of niraparib regardless of *BRCA* mutation status. This potent efficacy of niraparib may be related to its ability to concentrate in tumor relative to plasma, which might be particularly relevant in the setting of *BRCA*wt tumors.

### Differential killing of niraparib versus olaparib in *BRCA*wt versus *BRCA*mut models

The observation of greater TGI in *BRCA*wt models with niraparib vs. olaparib might relate to greater tumor exposure resulting from better cell membrane penetration achieved with niraparib. We therefore assessed the minimal efficacious dose (MED) of niraparib required to induce efficacious tumor growth inhibition in both *BRCA*mut and *BRCA*wt models.

**Figure 4:**
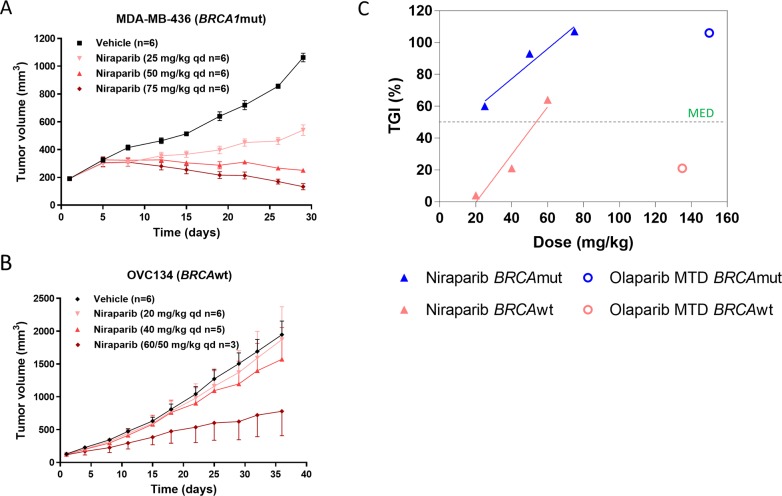
**(A & B)** Effect of different doses of niraparib on tumor volume in *BRCA1*mut MDA-MB-436 TNBC and *BRCA*wt OVC134 ovarian xenograft models. Niraparib was administered at 25, 50, and 75 mg/kg once daily for 28 days in MDA-MB-436 model (A) or at 20 and 40 mg/kg once daily, respectively, for 32 days and at 60 mg/kg once daily for 4 days and then at 50 mg/kg once daily for 28 days due to body weight loss observed in OVC134 model (B). One mouse from 40 mg/kg was excluded from final analysis due to extensive drug holiday applied to it, and 3 mice from 60 mg/kg treatment groups dead (B). **(C)** The correlation between percentage of TGI and doses used from Figure 4A & B and Figure 3B, 3C & 3D.

Niraparib was administered at 25, 50, or 75 mg/kg once daily for 28 days to mice bearing MDA-MB-436 *BRCA*mut tumors. Suppression of tumor growth was observed at all doses with TGIs of 60%, 93%, and 107%, respectively (Figure 4A). In the OVC 134 ovarian model, administration of 20, 40, or 60 mg/kg of niraparib once daily for 32 days resulted in 4%, 21%, and 64% TGI, respectively. Only the 60 mg/kg dose level (reduced to 50 mg/kg after 4 days of dosing due to body weight loss) was considered efficacious (Figure 4B), gauging upon the cutoff of ≥50% TGI. The MED of niraparib was estimated to be 10 mg/kg in the MDA-MB-436 *BRCA*mut model and 53 mg/kg in the OVC134 *BRCA*wt model by extending the dose-response curve obtained through linear regression fitting (Figure 4C). Notably, the MED of the *BRCA*wt OVC134 tumors (53 mg/kg) is 5 fold greater than the MED for the *BRCA*mut MDA-MB-436 tumors (10 mg/kg), suggesting, as expected, that tumors with different HR deficiency profiles have different sensitivities to PARPi. On the other hand, although olaparib was efficacious in the *BRCA*mut MDA-MB-436 model, it failed to induce a meaningful antitumor effect in the *BRCA*wt OVC134 model (21% TGI) at MTD (150 mg/kg/day) (Figure 4C), suggesting that high tumor exposure and cell membrane permeability of niraparib may contribute to its potent antitumor effect in *BRCA*wt tumors.

### Tumor response in a PDX model is improved when switching to niraparib from olaparib

**Figure 5:**
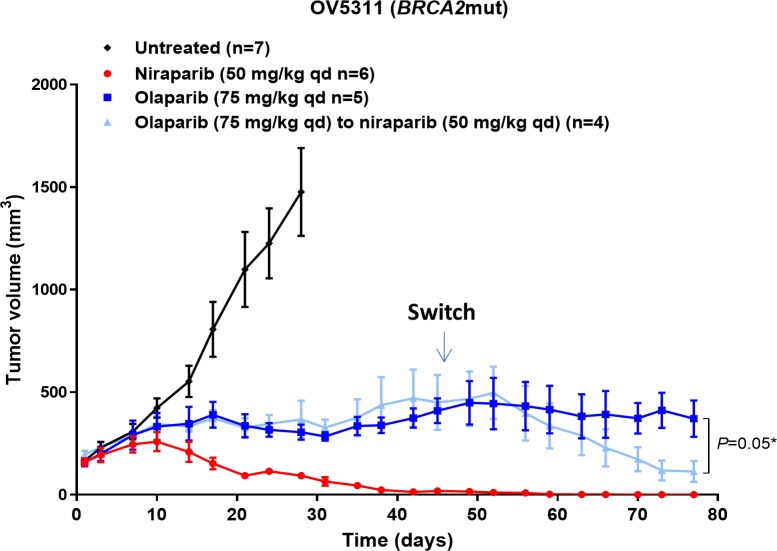
Effect of niraparib and olaparib on tumor volume in *BRCA2*mut OV5311 ovarian cancer PDX model Tumor volume of the OV5311 model treated with niraparib or olaparib at the maximum tolerated dose. Niraparib was administered at 75 mg/kg daily for 78 days. Olaparib was administered at 75 mg/kg once daily for 44 days and then 4 mice from this group were switched to 50 mg/kg of niraparib once daily for 34 days, while the remaining 5 mice continued with 75 mg/kg of olaparib.

An important question in cancer care is whether there will be any benefit in switching from one agent to another agent in the same drug class. Physicians face this question often, when drug efficacy is limited. To mimic this situation, we next tested the effect of sequential treatment with olaparib followed by niraparib in the *BRCA2*mut OV5311 ovarian cancer PDX model. When treated with niraparib or olaparib at 50 or 75 mg/kg qd, respectively, for 44 days, niraparib treatment resulted in complete tumor regression, whereas olaparib failed to induce tumor regression (Figure 5). On treatment day 45, mice in the olaparib group were randomized to two treatment groups, switching to 50 mg/kg of niraparib (4/9 mice with the average tumor size of 450 mm^3^), or remaining on the same olaparib treatment (5/9 mice with the average tumor size of 409 mm^3^). At the end of the treatment on day 77, significant tumor regression was observed in the animals switched from olaparib to niraparib treatment in comparison to the animals continuing treatment on olaparib (*P*=0.05) (Figure 5). This result shows that the tumor response improved upon switching from olaparib to niraparib, suggesting either a concentration dependent effect, or an additional mechanism of action for niraparib in comparison to olaparib, or possibly both.

### Niraparib is efficacious in an intracranial tumor model

**Figure 6:**
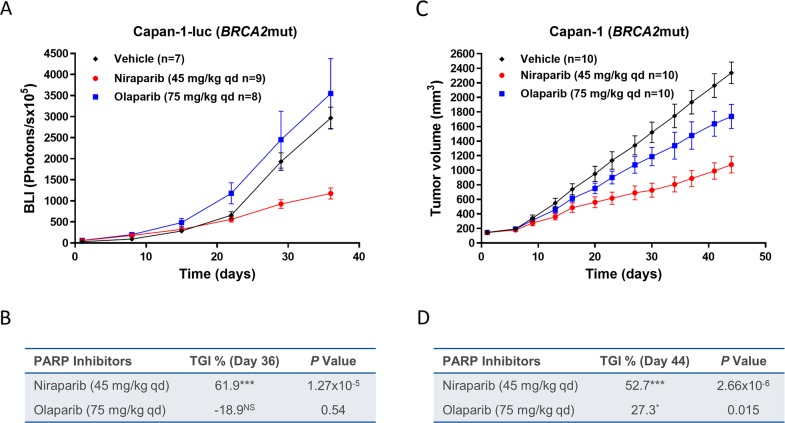
Effect of niraparib and olaparib on tumor growth in intracranial *BRCA2*mut Capan-1-luc or subcutaneous Capan-1 pancreatic cancer xenograft model **(A)** Tumor bioluminescent signals of the intracranial Capan-1-luc model treated with niraparib or olaparib. Niraparib or olaparib was administered at 45 or at 75 mg/kg daily, respectively, for 35 days. Two mice from control group were removed from data analysis since they were identified as the major outliers based on statistical analysis. One mouse from control or niraparib-treated group and two mice from olaparib-treated group died during treatment. **(B)** Table summarizing TGI and *P* value calculated by Student’s *t* test for niraparib or olaparib compared to vehicle on day 36, luc=luciferase; ^***^P< 0.001, NS=not significant. **(C)** Tumor growth of the subcutaneous Capan-1 model treated with niraparib or olaparib. Niraparib or olaparib was administered at 45 or at 75 mg/kg daily, respectively, for 44 days. **(D)** Table summarizing TGI and *P* value calculated by Student’s *t* test for niraparib or olaparib compared to vehicle on day 44, ^***^P< 0.001, ^*^P< 0.05.

To further understand if sustained brain exposure to niraparib could result in antitumor activity in brain tumor models, BALB/c nude mice were intracranially inoculated with the *BRCA2*mut Capan-1-luc tumor cells. The antitumor effects of daily niraparib treatment at 45 mg/kg were compared with 75 mg/kg qd of olaparib, with both drugs administered at MTD. After 35 days of treatment, niraparib resulted in 62% TGI compared to −19% TGI for olaparib (Figure 6A, 6B, & Supplementary Figure 2C). The antitumor effects of niraparib at 45 mg/kg qd and olaparib at 75 mg/kg qd were also tested in mice bearing subcutaneously inoculated Capan-1 tumor cells. Niraparib resulted in 53% TGI compared to 27% TGI for olaparib after 44 days of treatment (Figure 6C & 6D). None of the treatments induced significant body weight loss (data not shown). The differential anti-tumor effect of the two PARPi in the subcutaneous model suggested that this model maybe intrinsically more sensitive to niraparib. Notably, the niraparib efficacy in the intracranial model was similar to that in the subcutaneous model derived from the same cell line, suggesting that the brain exposure of niraparib is efficacious in this model. In comparison, the minimal effect of olaparib in the subcutaneous model was lost in the intracranial model, potentially due to its insufficient brain exposure.

### Megakaryocyte toxicity is a class effect of PARPi *in vitro*

Clinically, niraparib treatment can result in thrombocytopenia in some patients. In this study, we observed that niraparib, in contrast to olaparib, distributed to bone marrow, where platelets are generated by megakaryocytes. We therefore evaluated the toxicity of PARPi on megakaryocytes differentiation *in vitro*. CD34^+^ cells were harvested from normal human bone marrow and differentiated into megakaryocytes. In this model system, the first 7 days of culture are dominated by proliferation of early progenitor cells, with differentiation and maturation occurring typically between day 7 and 10. Niraparib and olaparib were incubated with the CD34^+^ cells at 3 different concentrations throughout the course of the 10-day study, with 5-fluorouracil (5-FU) treatment serving as a positive control to evaluate its toxicity to megakaryocytes. Both drugs inhibited CD34^+^ cell proliferation, differentiation and maturation with similar potency on day 7 (niraparib IC_50_ = 1.2 μM; olaparib IC_50_ = 1.6 μM) and day 10 (niraparib IC_50_ = 0.8 μM; olaparib IC_50_ = 1.3 μM), suggesting that the comparable toxicity to megakaryocyte lineage cells from both compounds, thus likely a class effect of PARPi (Supplementary Figure 5A & 5B). Greater bone marrow exposure associated with the high V_D_ for niraparib may explain the thrombocytopenia observed with this agent at higher doses, with the 76-fold higher exposure in tumor than bone marrow (Table 3) rationalizing the large therapeutic window observed clinically. Bone marrow exposure to olaparib was not observed in the same tumor model, consistent with the low tumor exposure relative to plasma potentially resulting from a low V_D_ observed in preclinical and clinical settings.

## DISCUSSION

This study compared niraparib and olaparib in terms of their pharmacokinetic properties and antitumor activities in several well characterized tumor xenograft models, and provides the first direct evidence that MTD administration of niraparib achieved higher exposures in tumor and some other tissues than olaparib. Consistent with its higher V_D_, niraparib tends to accumulate in tumor and other tissues rather than circulating in plasma, whereas olaparib tumor exposure is lower than plasma exposure. Niraparib also demonstrated significantly longer duration of exposure above efficacious concentration in the brain compared to olaparib. Together with the superior efficacy of niraparib observed in this study, our results demonstrate the importance of differential drug exposure and its association with antitumor activity, especially in *BRCA*wt and intracranial tumors.

Niraparib plasma AUC is also significantly higher than olaparib, even when olaparib is administered at higher daily dose, potentially due to the high bioavailability of niraparib. In addition, niraparib is not a substrate of the hepatic uptake transporters, OATP1B1 and OATP1B3, in particular, as well as the renal uptake transporters, such as OAT1, OAT3, and OCT2 (data not shown). Therefore, there is minimal, if any, involvement of transporters in clearance/elimination of niraparib. In contrast, olaparib inhibits the hepatic drug uptake transporters OATP1B1 and OCT1 as well as the renal uptake transporters OCT2 and OAT3 [44], which may affect clearance of olaparib.

Notably, these results clearly indicate that niraparib and olaparib, which have similar *in vitro* PARP catalytic inhibition potency and cytotoxicity in *BRCA*mut cells [22] as well as *in vivo* efficacy in *BRCA*mut xenograft model, are not equivalent with respect to their effectiveness on tumor growth inhibition, particularly in *BRCA*wt models. Niraparib demonstrated greater efficacy compared to olaparib *in vivo*, a phenotype that could, at least partially, be attributed to their different pharmacokinetic properties, i.e. V_D_ and cell permeability. This may also explain why niraparib has shown stronger activity in non-*BRCA*mut patients in clinical studies, as seen in the NOVA trial [23]. In this trial, niraparib treatment led to a 5.4-month improvement in the ovarian cancer patient without germline *BRCA* mutation. In comparison, olaparib delivered 1.9 months of PFS improvement in *BRCA*wt ovarian cancer patients in a Phase 2 clinical study (Study 19) [24]. Several previous *in vitro* studies have demonstrated that higher concentrations of PARPi are required to induce cell death in *BRCA*wt cells compared with *BRCA*mut cells [22, 45, 46]. Our results provide evidence that niraparib is effective in both OVC134 (*BRCA*wt) and MDA-MB-436 (*BRCA*mut) tumor models, with a higher MED in *BRCA*wt tumors, suggesting that HR deficient tumors without *BRCA* mutation may require higher tumor PARPi concentrations to be efficacious.

To better mimic the clinical dosing schedule of olaparib, our experimental dose was optimized from 100 mg/kg qd administered in MDA-MB-436 model to 67 mg/kg bid in OVC134 model. However, possibly related to its relatively lower tumor exposure, bid dosing of olaparib did not result in meaningful antitumor efficacy (21% TGI) in the *BRCA*wt OVC134 model. The total daily doses of olaparib at MTD with qd and bid dosing remain similar, suggesting that dose and schedule change did not improve therapeutic window.

Although niraparib tumor exposure has yet to be reported in patients, such data will be important for detailed interpretation of clinical performance. Pharmacokinetic modeling may also provide helpful information, especially when combined with observed clinical tumor concentration.

When attempted to correlate PARylation inhibition with antitumor effects in preclinical models, we noticed that inhibition of total PARylation is not a good biomarker for PARPi efficacy. Better assays need to be developed as tools for understanding PARPi activity in tumor models and clinical samples, such as PARP trapping assays or assays measuring substrate specific PARylation suitable for clinical sample analysis.

Even though both niraparib and olaparib are substrates of P-gp, the fact that niraparib can penetrate the BBB, where P-gp is highly expressed, suggests that niraparib might be able to overcome the efflux of P-gp potentially due to its high bio-membrane permeability. The relatively lower efflux rates compared to olaparib in P-gp and BCRP overexpressing cells observed in this study partially confirmed this hypothesis. More studies on specific mechanisms, such as directly measuring intracellular drug concentration, will provide more insights.

The brain exposure to niraparib was sustainable at the steady state in the tumor-free brain of OVC134 model, with ≈4 μM at C_max_ and ≈0.6 μM at trough. In contrast, although olaparib can penetrate the brain with ≈2 μM at C_max_, it was eliminated from the brain quickly as its concentration dropped to 0.1 μM at 2 hours post dose and below the limit of detection at 6 hours post dose. The differences of brain exposure to niraparib and olaparib are more evident by their dose-normalized AUC ratios, 100- and 34-fold, in the MDA-MB-436 and OVC134 models, respectively. Clinical testing of such agents in brain tumors largely relies on a compromised BBB, a phenomenon related to late stage disease. On the contrary, agents that cross a competent BBB are required to control early stage brain malignancy and prevent brain metastasis from certain types of lung and breast cancers. Even though ovarian cancer-related brain metastases are relatively uncommon, the incidence rate has been reported to range from 0.49% to 11.54% with an average of 2.55% [47]. The therapeutic brain exposure to niraparib thus differentiates it from olaparib in preclinical models and merit to test niraparib in clinic against primary brain malignancy and clinical indications with high risk of brain metastases, such as lung and breast cancers. In addition, the ability of niraparib to penetrate the BBB not only reveals new opportunities for clinical development in brain malignancies, but also highlights the possibility of overcoming P-gp-induced drug resistance, as P-gp is highly expressed at BBB.

The synthetic lethality between PARPi and HR deficiency in tumor cells has been well established, but the lack of clinically suitable biomarker for identifying HR deficiency posts a challenge to clinical development of PARPi. Potential companion diagnostic tests have been developed, such as the myChoice HRD test (Myriad Genetics) and the fLOH test (Foundation Medicines), using genomic instability as biomarker for HR deficiency. However, these tests cannot identify all HR deficient patients, such as the ones with functional HR deficient tumors. Therefore, other approaches were also utilized to enrich HR deficient population in clinical development. For example, platinum response may enrich PARPi sensitive OC population, as HR deficient tumors and nucleotide excision repair deficient tumors are both sensitive to platinum treatment [48, 49].

In the NOVA trial (NCT01847274), the most common treatment-emergent adverse event leading to dose modification was thrombocytopenia, which was reversible and typically occurred during the first month of treatment. Dose modification of niraparib was shown to reduce the rate of thrombocytopenia with no reduction in efficacy [50]. While *in vitro* results from this study, consistent with what reported recently [51], suggested PARPi toxicity on megakaryocyte lineage cells, olaparib treatment induced thrombocytopenia and other hematological toxicities were not observed to the same extent as were seen with niraparib in clinical trials. One potential explanation could be the difference in tissue distribution including bone marrow exposure. As observed in our preclinical PK analysis, olaparib was undetectable in bone marrow, consistent with its low tumor exposure in preclinical models and low V_D_. While niraparib was detectable in bone marrow, which however was 76-fold lower than that in tumor, suggesting a large therapeutic window.

In summary, this study demonstrated, for the first time, the higher tumor and tissue exposure to niraparib compared to olaparib and associated correlation with enhanced antitumor activity in the xenograft animal models, especially in some *BRCA*wt tumors evaluated. More preclinical and clinical studies are clearly warranted to comprehend the potential of niraparib to benefit the patients with a variety of cancers.

## MATERIALS AND METHODS

### Chemicals

Niraparib was synthesized in-house as described previously [52]. Olaparib was obtained from SelleckChem (Houston, TX). Niraparib was formulated in 0.5% methyl cellulose (Sigma-Aldrich, Shanghai, China) and olaparib was formulated in 10% DMSO (Sigma-Aldrich, Shanghai, China), 10% hydroxypropyl-β-cyclodextrin (Zibo Qianhui Biotechnology Co. Ltd., Shangdong, China) in PBS pH7.4 (GE Healthcare Life Science, Logan, Utah).

### Cell culture

A2780, MDA-MB-436, Capan-1 and Capan-1-luc cells were maintained *in vitro* as a monocultures at 37°C in an atmosphere of 5% CO_2_ in air using RPMI1640 medium supplemented with 20% heat inactivated fetal bovine serum; L-15 medium supplemented with 10% fetal bovine serum, 10μg/mL insulin and 16μg/mL L-glutathione; IMDM medium supplemented with 20% FBS, 100 U/mL penicillin and 100 μg/mL streptomycin, and 2 mM L-glutamine, respectively. The A2780 or MDA-MB-436 tumor cells were routinely sub-cultured twice weekly by 10μg/mL Insulin treatment or without any treatment. The Capan-1-luc cells were started at 5×10^5^ viable cells/mL and sub-cultured at 1×10^6^ cells/mL. Fresh growth medium (20% to 30% by volume) was added every 2 to 3 days. The cells growing in an exponential growth phase were harvested and counted for tumor inoculation.

### *In vitro* A2780 cell cytotoxicity assays

A2780 cells were seeded in triplicate at densities of 10^5^ per well for cytotoxicity assays in 96-well flat-bottomed plates with the recommended culture medium. Cells were then incubated with either olaparib or niraparib for 6 days in recommended growth medium supplemented with 10% (v/v) FBS. Cell viability was assessed using MTT assays as previously described [53].

### *In vivo* PK and efficacy studies

PK and efficacy studies in the orthotopic MDA-MB-436 human cell line-derived breast cancer model, subcutaneous A2780 and OVCAR3 human cell line-derived ovarian cancer models, and subcutaneous OV5308 and OV5311 patient-derived ovarian cancer xenograft models were conducted at Crown Bioscience (Taicang, China or San Diego, USA). PK and efficacy studies in the OVC134 and OVC527 patient-derived ovarian cancer xenograft models and subcutaneous Capan-1 and intracranial Capan-1-luc human cell line derived pancreatic cancer models were performed at Pharmaron, Inc. (Beijing, China). PK studies were conducted with 3 mice at each timepoints. For efficacy studies, typically 6 to 10 mice were randomized to each group. The protocol and any amendment(s) or procedures involving the care and use of animals in these studies were reviewed and approved by the Institutional Animal Care and Use Committee (IACUC) of Pharmaron or CrownBio prior to conduct. During the study, the care and use of animals was conducted in accordance with the regulations of the Association for Assessment and Accreditation of Laboratory Animal Care (AAALAC).

Seven- to 9-week-old female NOD/SCID mice (Beijing HFK Bio-Technology Co. Ltd., Beijing, China or Shanghai Lingchang Bio-Technology Co. Ltd., Shanghai, China) were inoculated at the right mammary fat pad with the MDA-MB-436 tumor cells (1 × 10^7^) in 0.2 mL of phosphate-buffered saline (PBS) mixed with matrigel (1:1) for tumor development. For the A2780 or OVCAR3 tumor development, 6- to 8-week-old or 8- to 9-week-old female BALB/c Nude mice (Shanghai SIPPR-Bk Lab Animal Co. Ltd., Shanghai, China) were inoculated subcutaneously at the right flank with 5 × 10^6^ tumor cells in 0.1 mL of PBS (1:1 mixed with matrigel) or with 1 × 10^7^ tumor cells in 0.1 mL of PBS (1:1 mixed with matrigel). 6- to 8-week-old female NOD/SCID mice (Envigo Laboratories, USA) were inoculated at the rear flank with the OV5311 tumor cells (3 × 10^5^) in 0.2 mL of PBS mixed with Cultrex ECM (1:1) or with OV5308 tumor cells (9 × 10^4^) in 0.2 mL of PBS mixed with Cultrex ECM (1:1) for tumor development. 6- to 8-week-old female BALB/c nude mice (Beijing AniKeeper Biotech Co. Ltd., Beijing, China) were inoculated subcutaneously on the right flank with a tumor fragment (2 mm × 2 mm × 2 mm) of the OVC134 or OVC527 human primary ovarian cancer for tumor development. For the Capan-1 subcutaneous tumor model development, 6- to 8-week-old female BALB/c Nude mice were inoculated subcutaneously at the right flank with 4 × 10^6^ tumor cells in 0.1 mL of IMDM without serum (1:1 mixed with matrigel). For the Capan-1-luc tumor development, mice were anesthetized by intraperitoneal (IP) injection of ketamine/xylazine (90-120/5-10mg/kg). The skins over the coronal and sagittal sutures were sterilized with iodine followed by alcohol. An incision of 0.5 cm was made along the skin over the midline to expose coronal and sagittal suture junctions. An inoculum of 2 × 10^5^ Capan-1-luc tumor cells (in 2 μL IMDM) were injected into the right forebrain by positioning the needle at 2.0 mm lateral to the sagittal suture, 1.0 mm inferior to coronal suture with the injection depth precisely controlled at 3.0 mm. The injection was slowly proceeding over a one-minute period.

After tumor cell inoculation, the animals were checked daily for morbidity and mortality. At the time of routine monitoring, the animals were checked for any effects of tumor growth and treatments on normal behavior such as mobility, visual estimation of food and water consumption, body weight gain/loss (body weights were measured 2 to 3 times per week throughout the study), eye/hair matting and any other abnormal effect. Any mortality and/or abnormal clinical signs were recorded.

Tumor volumes were measured 3 times weekly in 2 dimensions using a caliper, and the volume was expressed in mm^3^ using the formula: V = 0.5 a × b^2^ where a and b were the long and short diameters of the tumor, respectively. The entire procedures of dosing as well as tumor and body weight measurement were conducted in a Laminar Flow Cabinet. For the Capan-1-luc tumors, mice were injected intraperitoneally with D-luciferin of 7.5 mg/mL (at 10μl/g BW) and anesthetized with isoflurane inhalation. At 10 minutes after the luciferin injection, mice were imaged with an *In Vivo* Imaging System (IVIS, Xenogen/Caliper, USA). Bioluminescent signals (photons/s) from the region of interest (ROI) were quantified and used as an indicator of tumor growth. Tumor bioluminescent signals were conducted once a week. For PK studies, the treatment was started when the average tumor size reached approximately 320 to 360 mm^3^. For the Capan-1-luc intracranial efficacy study, the treatment was started when the mean bioluminescent signal reaches approximately 5.5 × 10^6^ photons/s. For all other efficacy studies, the treatment was started when the average tumor size reached approximately 50 to 200 mm^3^. The tumor size was used for the calculation of tumor growth inhibition (TGI) = [1 - (T_1_-T_0_)/(C_1_-C_0_)] × 100, where C_1_= mean tumor volume of control mice at time t, T_1_ = mean tumor volume of treated mice at time t, C_0_ = mean tumor volume of control mice at time 0, and T_0_ = mean tumor volume of treated mice at time 0.

Niraparib, olaparib, or vehicle (0.5% methyl cellulose) was administered orally once daily in the MDA-MB-436 PK, as well as A2780, OV5311, OVCAR3, Capan-1 and Capan-1-luc efficacy studies. Olaparib was administered orally twice daily in both OVC134 PK and efficacy studies, as well as the MDA-MB-436, OV5308, OVC527 efficacy studies as per Institutional Animal Care and Use Committee guidelines.

### PK analysis of PARPi

Frozen tumor, brain, bone marrow, muscle and plasma samples were shipped to Charles River Laboratories (Agilux Laboratories, Inc, Worcester, MA) for bioanalytical sample analysis. Internal standard stock solution was prepared at 1 mg/mL in dimethyl sulfoxide. Internal standard spiking solution was prepared at 100 ng/mL in 80:20 (v:v) water:acetonitrile. CD-1 mouse plasma (K_2_EDTA), pooled mixed gender, was purchased from BioChemed Services (Winchester, VA). The blank matrix was tested for interference at the retention time and mass transition of the analytes and internal standard, and was found to be free of significant interference. For niraparib, calibration standards were prepared on the day of sample extraction at concentrations of 1.00, 2.50, 5.00, 20.0, 50.0, 100, 250, and 500 ng/mL niraparib in blank mouse plasma. Quality control samples were prepared at concentrations of 5.00, 50.0, and 250 ng/mL niraparib in blank mouse plasma. For olaparib, calibration standards were prepared on the day of sample extraction at concentrations of 1.00, 2.00, 5.00, 10.0, 50.0, 100, 500 and 1000 ng/mL olaparib in blank mouse plasma. Quality control samples were prepared at concentrations of 5.00, 50.0 and 500 ng/mL olaparib in blank mouse plasma. Plasma samples were not diluted prior to extraction. Tumor, muscle and brain samples were prepared for analysis by homogenization (on ice). After the samples were weighed, for every 1 g of tissue, 9 mL of 80:20 (v:v) water:acetonitrile was added to the sample. The samples were homogenized using a hand-held homogenizer. The dilution factor for the sample was 10. After homogenization, each sample was diluted by a factor of 5 with blank mouse plasma, K_2_EDTA (10 μL of homogenate into 40 μL of plasma). The total dilution factor for the sample was 50. Bone marrow samples were diluted by a factor of 5 with blank mouse plasma, K_2_EDTA (10 μL of sample into 40 μL of plasma). The dilution factor for the sample was 5. Niraparib and olaparib in the supernatants from plasma, brain, bone marrow, muscle and tumor samples were quantitatively determined using a qualified ultra-performance liquid chromatography with tandem mass spectrometric detection (UPLC-MS/MS) (Agilent 1290 binary pump and a PAL HTC-XT autosampler, Applied Biosystems Sciex API 6500+ mass spectrometer). A Waters BEH C18 column (50 × 2.1 mm; 1.7 μm) was used and maintained at 50°C during analysis. The instrument was equipped with an electrospray ionization source in positive-ion mode and the analytes were monitored in the multiple-reaction-monitoring scan mode. The calibration curve range for niraparib was 1-500 ng/mL, and that for olaparib was 1-1000 ng/mL. The software application used to acquire and process the data for this study was Analyst^®^ Version 1.6 (Applied Biosystems Sciex, Framingham, MA).

PK parameters for niraparib and olaparib were calculated using noncompartmental methods. The following parameters were determined in each animal, where possible, using Phoenix WinNonlin v7.0:

C_max_ maximum observed plasma concentration that a drug achieves in a specified compartment.

t_max_ time of occurrence of C_max_.

AUC_0-24h_ area under the plasma concentration versus time curve within time span 0 to 24 hours of post dose (C_24hr_), calculated by the linear trapezoidal rule.

Plasma concentrations were provided in units of ng/mL. PK parameters were determined using nominal sampling times. Dose normalized AUC_0-last_was derived after correcting concentrations to molar units using molecular weights of 320.39 and 434.46 Da for niraparib and olaparib, respectively, and then divided by total amount of drug used per 24 hours for niraparib or 12 hours for olaparib.

Plasma concentrations below the lower limit of quantification of the assay (BLQ) were set zero if they occurred before the first measurable time point and were treated as missing if they occurred after the last measurable time point or in between 2 measurable time points.

### *In vitro* liquid culture system to assess human megakaryocyte-specific development

*In vitro* human megakaryocyte differentiation was assessed using a liquid culture system in which the human bone marrow CD34^+^ cells (lot # 0150114, ReachBio) derived from normal bone marrow were used. X-Vivo 15 (Lonza) containing 100 ng/mL SCF (Peprotech) and 50 ng/mL Tpo was used to drive CD34^+^ cells proliferation and differentiation into megakaryocyte lineage cells. Test drugs were incubated with CD34^+^ cell for either 7 days or 10 days. The megakaryocyte lineage cells characterized by an ability to exclude DAPI were analyzed by flow cytometry. The percentage of megakaryocyte lineage cells in the total CD34^+^ cells were then calculated. To determine the IC_50_ values, XLFit was used. The dose-response data for the relevant experimental conditions were tabulated and then processed using curve-fitting algorithm 205 provided in XLFit.

### *In vitro* human BCRP and MDR1 efflux (ABC) transporters assay

The monolayer assays were performed using parental and BCRP or MDR1 transfected MDCKII cell monolayers. MDCKII, MDCKII-BCRP and MDCKII-MDR1 cells were cultured in Dulbecco’s Modified Eagle’s Medium with 4.5 g/L glucose (DMEM) supplemented with 10% (v/v) fetal bovine serum (FBS) at 37 ± 1°C in an atmosphere of 95:5 air:CO_2_ in cell culture flasks prior to seeding into 24-transwell inserts (Corning). Transfected and parental MDCKII cells were cultured on the inserts with 100 μL medium per well on the apical side and 25 mL in a single-well receiver tray for all 24 wells on the basolateral side, for 96 hours. Medium was changed 24 hours before the experiment.

Permeability incubations were carried out in Hank’s Buffered Salt Solution (HBSS) at 37 ± 1°C. Apical to basolateral permeability of Lucifer Yellow (LY) was assessed as a low permeability control, LY was also incubated in the presence of the test articles (at the highest testing concentration: 100 μM) in order to assess the effect of the compounds on the monolayer integrity. LY samples (100 μL from basolateral wells) were analyzed by measuring fluorescence with the following wavelengths: excitation – 430 nm, emission – 520 nm. Trans-epithelial electric resistance (TEER) of each well was measured to confirm the confluency of the monolayers after the experiments.

The assays were conducted according to the actual SOLVO MDCKII-BCRP and MDCKII-MDR1 monolayer assay protocols (SOP).

Bidirectional transport of niraparib and olaparib was determined through control (parental or mock-transfected), MDCKII-BCRP and MDCKII-MDR1 cell monolayers. Cells were pre-incubated in assay buffer for 10 minutes to allow cells adjusting to the medium. Assay buffer containing the test articles (separately) at three concentrations (1 μM, 10 μM and 100 μM) was then added to the appropriate apical (100 μL) or basolateral (600 μL) chamber. The final concentration of the organic solvents in the incubations did not exceed 0.5% (v/v). The prazosin or digoxin efflux ratio was determined as a positive control for BCRP or MDR1 function, respectively.

After incubation at 37 ± 1°C for 120 minutes, aliquots (50 μL) were taken from the receiver chambers to determine the amount of translocated olaparib or niraparib and controls. Samples (50 μL) were also taken from the helper plate and donor chambers before and after incubation, respectively to determine the initial concentrations (C0) and recovery (R) of the test compounds and the controls. Bidirectional transport of the test articles in control, MDCKII-BCRP and MDCKII-MDR1 cells was determined by LC-MS/MS.

### poly(ADP-ribose) (PAR) formation pharmacodynamic study

Tumor was quickly collected, cut into pieces, weighed, snap frozen in liquid nitrogen and stored at −80°C. Cell extracts were prepared from one piece of frozen tumors according to HT PARP *in vivo* Pharmacodynamic Assay II protocol (Trevigen, Gaithersburg, MD) at Crown Bioscience or at Pharmaron, Inc. Net PAR levels were determined in the MDA-MB-436 tumors or OVC134 tumors collected immediately following 5 days or 2 days of treatment with either control, niraparib, or olaparib. Experimental samples and freshly made PAR standards were assayed in duplicate. The assay was repeated once for the niraparib-treated MDA-MB-436 tumors. PAR concentrations in the experimental samples were calculated using a standard curve, and PAR levels were normalized to the amount of total protein and reported as pg PAR per 100 μg of total protein. Concentration of the total protein in the extract was measured using the BCA Assay (Thermo Fisher Scientific, Waltham, MA).

### Statistical analysis

Differences between values obtained in cell lines and tumors treated with different experimental conditions were determined using the Student’s *t* test on GraphPad Prism 7 (GraphPad Software). *P*< 0.05 was considered statistically significant.

## SUPPLEMENTARY MATERIALS FIGURES AND TABLE


