# Supplementary material for: A comparative pharmacokinetic study of PARP inhibitors demonstrates favorable properties for niraparib efficacy in preclinical tumor models
Source: Oncotarget. 2018 Dec 14;9(98):37080–96. doi: 10.18632/oncotarget.26354 (PMC6324689; doi:10.18632/oncotarget.26354)
Supplement: Supplementary file 1 [file oncotarget-09-37080-s001.pdf]

A comparative pharmacokinetic study of PARP inhibitors demonstrates favorable properties for niraparib efficacy in preclinical tumor models

SUPPLEMENTARY MATERIALS

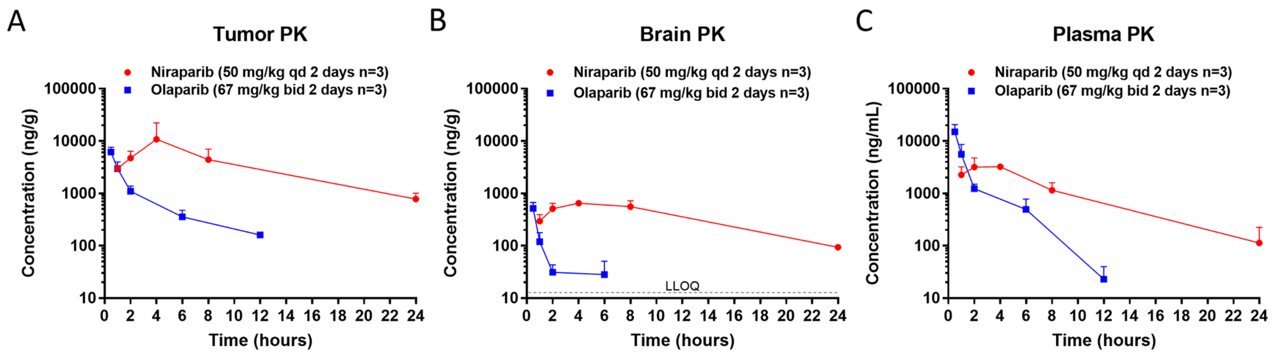

Supplementary Figure 1: Steady state pharmacokinetics (PK) of niraparib and olaparib in tumor, brain, and plasma. **(A)** Tumor, **(B)** Brain, and **(C)** Plasma PK in the *BRCA1*wt OVC134 ovarian cancer PDX model treated with niraparib or olaparib at the maximum tolerated dose. Niraparib or olaparib was administered either at 50 mg/kg qd or at 67 mg/kg bid for 2 days, and samples were collected on the last day of treatment at 1, 2, 4, 8, and 24 hours post dose for niraparib and at 0.5, 1, 2, 6, and 12 hours post dose for olaparib. LLOQ=lower limit of quantification.

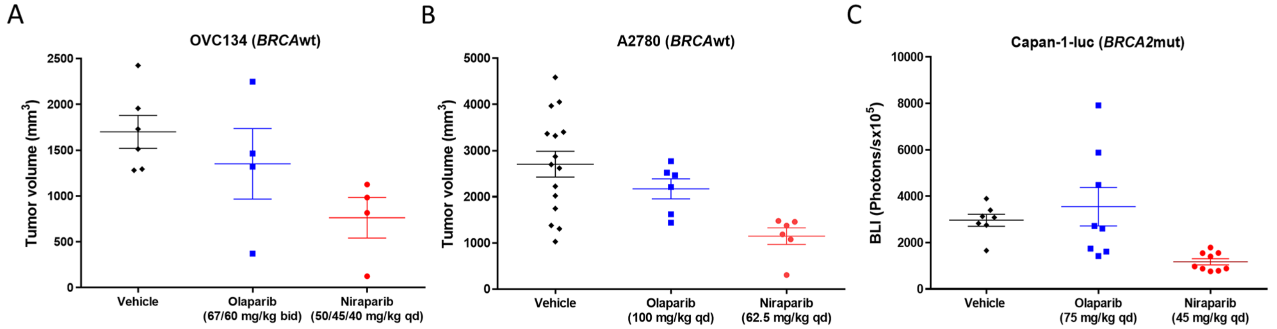

Supplementary Figure 2: Effect of niraparib and olaparib on tumor volume on the last day of treatment in individual mouse. **(A)** Scatter plot of individual tumor volume on day 40 from a *BRCA*wt OVC134 ovarian cancer PDX model. **(B)** Scatter plot of individual tumor volume on day 9 from *BRCA*wt A2780 ovarian cancer cell line-derived xenograft model. **(C)** Scatter plot of individual tumor bioluminescent signal on day 36 from intracranial *BRCA2*mut Capan-1-luc pancreatic cancer xenograft model.

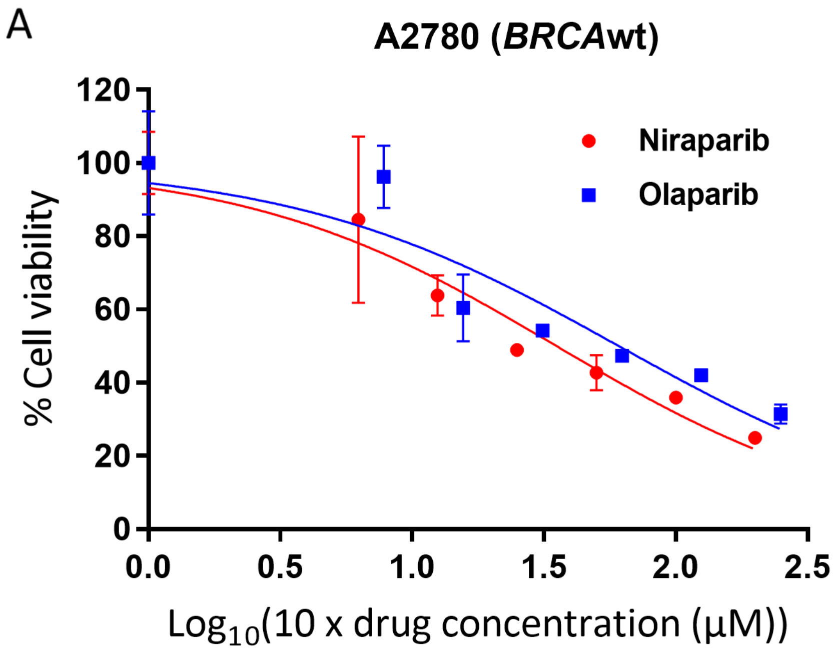

B

PARP Inhibitors	IC_{50} (μM)	*P* Value
Niraparib	2.6±0.93	0.77
Olaparib	5.11±0.98	

Supplementary Figure 3: (A) Dose-response curves of cell viability in A2780 cells treated with niraparib and olaparib for 6 days, assessed by MTT assays. Data are normalized to DMSO-treated cells. Error bars represent SD of triplicate replicates. **(B)** Table summarizing calculated IC_{50} shown as μM for each drug and *P* value calculated by Student's *t* test to compare niraparib and olaparib.

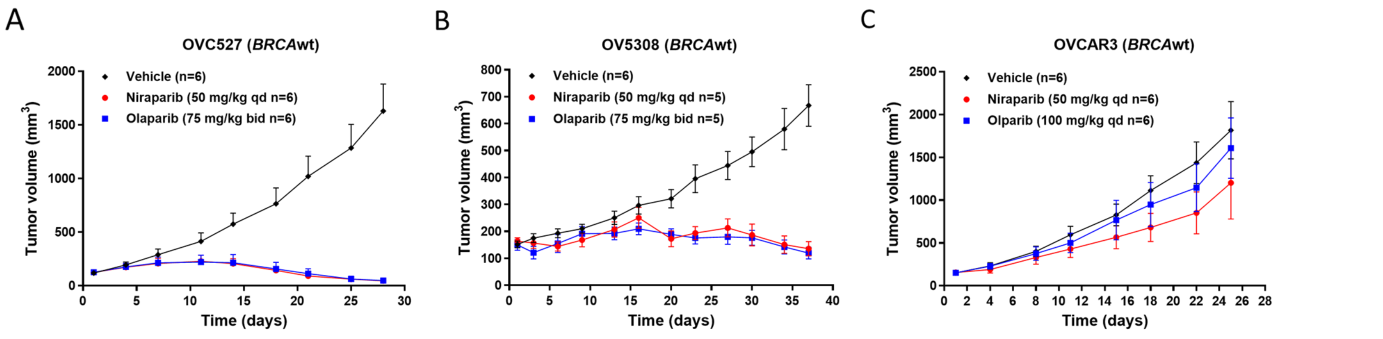

Supplementary Figure 4: Effect of niraparib and olaparib on tumor volume in *BRCA*wt OVC527, OV5308 ovarian cancer PDX models, and *BRCA*wt OVCAR3 ovarian cancer CDX model. (A) Tumor volume of the OVC527 model treated with niraparib or olaparib at the maximum tolerated dose, 50 mg/kg daily or 75 mg/kg twice daily, for 28 days. **(B)** Tumor growth of the OV5308 model treated with niraparib or olaparib at the maximum tolerated dose, 50 mg/kg daily or 75 mg/kg twice daily for 37 days. One mouse from niraparib- or olaparib-treated group died. **(C)** Tumor growth of the OVCAR3 model treated with niraparib or olaparib at 50 mg/kg or 75 mg/kg daily for 25 days.

A

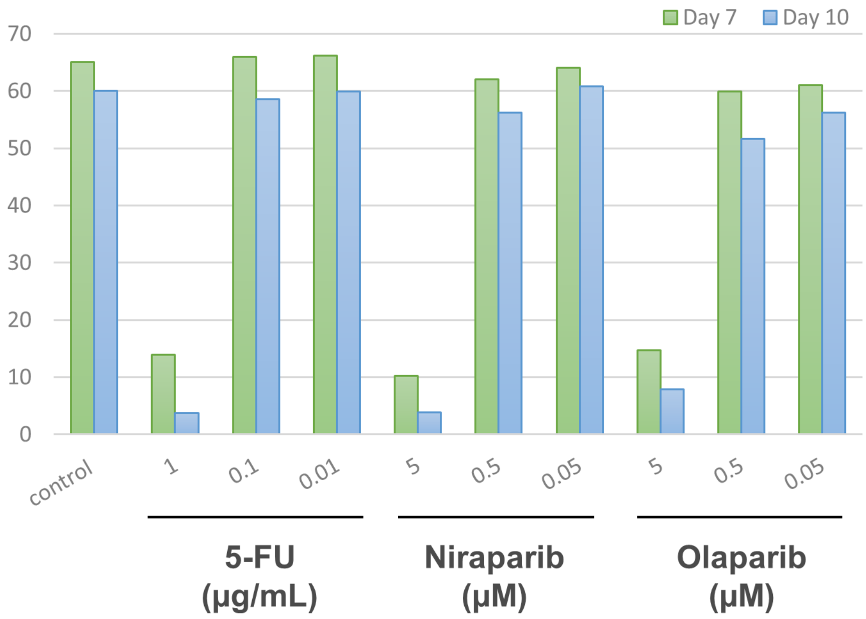

B

PARP Inhibitors	IC_{50} (μM) Day 7 (Proliferation)	IC_{50} (μM) Day 10 (Differentiation & maturation)
Niraparib	1.2	0.8
Olaparib	1.6	1.3

Supplementary Figure 5: Effect of niraparib and olaparib on megakaryocyte lineage cells proliferation as well as differentiation and maturation. **(A)** Dose response of niraparib and olaparib on megakaryocyte-lineage cells proliferation was evaluated after 7 days of treatment; differentiation and maturation was assessed after 10-day treatment in $CD34^+$ cells. The percentage of megakaryocyte-lineage cells in total $CD34^+$ cells was calculated and plotted. **(B)** Calculated IC_{50} shown as μM for each drug at day 7 and day 10, respectively.

Supplementary Table 1: Bidirectional Permeability and Net Efflux Ratio Values for Niraparib and Olaparib Across MDCKII-BCRP and MDCKII-MDR1 Monolayers

PARP inhibitors	Concentration (μM)	MDCKII-BCRP cells P_{app} A-B (× 10^{-6} cm/s)	MDCKII-BCRP cells P_{app} B-A (× 10^{-6} cm/s)	MDCKII-BCRP cells Net ER	MDCKII-MDR1 cells P_{app} A-B (× 10^{-6} cm/s)	MDCKII-MDR1 cells P_{app} B-A (× 10^{-6} cm/s)	MDCKII-MDR1 cells Net ER
Niraparib	100	4.10 ± 0.13	43.07 ± 2.62	9.58 ± 1.70	0.10 ± 0.01	2.66 ± 0.14	24.61 ± 5.32
	10	2.27 ± 0.70	59.59 ± 3.65	24.73 ± 6.47	1.32 ± 0.29	51.22 ± 1.43	37.71 ± 8.95
	1	4.83 ± 2.29	52.15 ± 3.09	8.86 ± 3.28	5.33 ± 3.51	54.26 ± 3.03	8.82 ± 4.94
Olaparib	100	0.90 ± 0.22	46.77 ± 3.40	50.54 ± 13.65	0.70 ± 0.05	57.50 ± 4.71	80.02 ± 5.98
	10	0.59 ± 0.33	44.14 ± 2.65	71.22 ± 14.97	0.51 ± 0.12	44.96 ± 6.41	82.56 ± 6.13
	1	1.12 ± 1.04	41.43 ± 4.35	32.97 ± 9.58	1.70 ± 0.69	46.45 ± 5.14	22.84 ± 2.65

ER=efflux ratio.
